# *Cyclin D1 G870A* Polymorphism: Relation to the Risk of ALL Development, Prognosis Impact, and Methotrexate Cytotoxicity

**DOI:** 10.31557/APJCP.2020.21.10.2941

**Published:** 2020-10

**Authors:** Nadia El Menshawy, Ahmed B El Marghany, Mohamed M Sarhan, Doaa A Aladle

**Affiliations:** 1 *Hematology Unit, Department of Clinical Pathology, Mansoura University, Egypt. *; 2 *Department of Pediatric, Mansoura University, Egypt. *

**Keywords:** ALL, Cyclin D1, CCND1, polymorphism, methotrexate

## Abstract

**Background:**

Cyclin D1 (CCND1) regulates cell cycle progression during the late G1 and S phase and takes part in methotrexate metabolism. It was hypothesized that *CCND1* gene polymorphism affects acute lymphoblastic leukemia (ALL) development, prognosis and may relate to methotrexate cytotoxicity.

**Subjects and methods:**

This study included 50 ALL patients and 50 healthy controls, *CCND1 G870A* polymorphism was studied in all items using polymerase chain reaction-restriction fragment length polymorphism (PCR-RFLP) and evaluated methotrexate cytotoxicity for ALL patients using liver function tests before and after methotrexate treatment. We followed up patients for one year to determine disease-free survival (DFS) and overall survival (OS) and its relation to the CCND1 genotype.

**Results:**

We found that AA genotype and A allele have a higher risk of developing ALL compared to the control group. Additionally, we found no notable association between CCND1 variant and methotrexate cytotoxicity and no role of *CCND1* polymorphism in ALL prognosis.

**Conclusion:**

Our results suggested that C*CND1 G870A* polymorphism is associated with a high risk of ALL development. However, it has no role in ALL prognosis or methotrexate cytotoxicity.

## Introduction

Childhood acute lymphoblastic leukemia (ALL) represents one of the most common tumors in children with an increased incidence of morbidity and mortality. Genetic susceptibility and exposure to environmental factors play an important role in leukemogenesis (Metayer et al., 2016)

Nowadays, Pharmacogenomics tests, including Genome-wide association studies (GWAS), have a direct identification role in genetic alterations, treatment efficacy, cytotoxicity, prognosis, etc. However, the current knowledge gives little data about how genomic determinants can affect ALL susceptibility, chemotherapy, and chance of relapse (Wu and Li, 2018). 

Methotrexate (MTX) forms the backbone of maintenance cycles in childhood acute lymphoblastic leukemia (ALL) chemotherapy, including interim maintenance (Mandal et al., 2020). Major adversities include cytopenias, mucositis, liver toxicity, renal toxicity and skin toxicity. These toxicities enquire dose modification or omission (burn out) MTX-related toxicities may be determined by several factors like dose, duration, genetic susceptibility and risk factors (Vaishnavi et al ., 2018).

Cyclin D1 (CCND1) is an essential regulator protein of the eukaryotic cell cycle. CCND1 acts mainly during the G1 phase and forms a complex with CDK4 or CDK6 in the mid to late G1 phase. This complex, activated by CDK-activating kinase, will cause phosphorylation of retinoblastoma protein (pRb). Phosphorylated Rb liberates transcription factors like E2F, which is responsible for gene transcription required for entry into the S phase of the cell cycle (Xue et al., 2015). Overexpression of *CCND1 *unsettles normal cell cycle process and probably stimulate the development and progression of childhood ALL (Kim andDiehl, 2009),colorectal cancer (Xie et al., 2017),cancer risk (Thakur et al., 2018).

A lot of research has been published regarding the effects of genetic polymorphisms on methotrexate (MTX)-induced toxicity and efficacy where it is a pivotal drug in the different treatment protocols, both at low and high doses. MTX acts on a variety of target enzymes in the folates cycle, as well as being transported out and into of the cell by several transmembrane proteins (Gervasin and Mota-zamorano., 2019).

Methotrexate (MTX), a folic acid antagonist, is a cornerstone in ALL treatment’s maintenance phase. One of MTX mechanisms is that it acts through competitive inhibition of dihydrofolate reductase (DHFR) regulated by CCND1 through increased E2F and thus leads to resistance to methotrexate (MTX) or interfere with its cytotoxicity. This leads to reducing the formation of tetrahydrofolate from dihydrofolate, resulting in the lack of the folate coenzymes leading to impairment of DNA and RNA synthesis (Li et al., 2016).

Several gene polymorphism have been investigated concerning susceptibility to ALL including SNPs of carcinogen metabolism genes, folate metabolism genes, DNA repair genes, regulators of lymphoid cell differentiation, tumor suppressors ,transcriptional factors and chemokines, in addition ,various SNPs participating in the metabolism of antileukemic agents inducing therapy toxicity , discontinuation of therapy with risk of relapse (Kampouraki et al.,2020).

Several studies have revealed that populations with the AA genotype had a significant risk of childhood ALL (Bedewy et al., 2013). Other studies reported the poor outcomes of youth ALL in the 870A allel. Furthermore, studies have shown the relation between methotrexate resistance or toxicity and the studied polymorphism (Xue et al., 2015). To the best of our knowledge, no study has linked the relation between childhood ALL susceptibility, Methotrexate resistance or toxicity, and the chance of relapse in one study. 

## Materials and Methods

This study included 50 patients with acute lymphoblastic leukemia aged from one year to 18 years (32 males and 18 females) diagnosed by peripheral blood and bone marrow morphology, immunophenotyping, and cytogenetic analysis. Also, 50 healthy (26 male and 24 female) subjects matched for age and sex served as the control group. All subjects’ parents in this study gave written informed consent before being included in the study. 

Thirty-two patients with B-ALL received low dose methotrexate, and 18 patients with T-ALL received high dose methotrexate. The study was conducted in the oncology center, Mansoura University, between April 2016 and October 2017. This study was ethically approved by Mansoura faculty of medicine Institutional research board (IRB), Mansoura University. 

*Genotyping of CCDN1 G870A gene*

Genotyping of the *CCDN1 G870A *gene was performed using the polymerase chain reaction-restriction fragment length polymorphism (PCR-RFLP) method. First of all, DNA was extracted, it was isolated from EDTA-blood samples by GeneJETTM Whole Blood Genomic DNA Purification Mini Kits from Thermo Scientific (lot no k0781, Lithuania, EU) according to manufacturer guideline. Then, analysis of genetic polymorphism using PCR-RFLP technique by two steps: first amplification of extracted genomic DNA that was done by using PCR via thermal cycler (ARKTIK Thermal Cycler, Thermo Scientific Co.) using a reverse primer (CCND1R-21): TTTCCGTGGCACTAGGTGTC and Forward primer (CCND1F-22) AGTTCATTTCCAATCCGCCC (BIOSEARCH Tech, Denmark).

**Figure 1:**
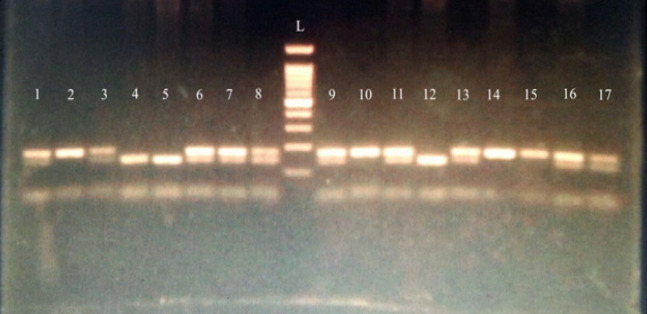
Agarose Gel (2%) Electrophoresis for RFLP Digested PCR Products. Lanes 2,10,14,15 represents restricted PCR product of an ALL patient with AA genotype (175 bp). Lanes 1,3,6,7,8,9,11,13,16,17 represent restricted fragments of PCR products of ALL patients with AG genotype (175,141 bp). Lanes 4, 5,12 represent restricted PCR products of ALL patients with GG genotype (141bp). Lane (L) represents 100bp ladder base pair marker

PCR was carried as follows: 12.5 µl MyTaqTM Red Master Mix (2X) (BioLine Ltd, UK). 1µl DNA template, forward and reverse primers 0.1µl each and 11.3µl nuclease-free water with the final volume of 25µl. PCR condition consisted of an initial denaturation of 95°C for 1 min, followed by 35 cycles of 94°C for 30 sec, 60°C for 30 sec, and 72°C for 30 sec, with a final extension of 72°C for 10 min. The PCR product of 212 bp was detected by agarose gel electrophoresis using 2% agar. Second, the PCR products were then digested with MspI (New England BioLabs Inc, lot no 0551507, UK). The digested PCR products were electrophoresed using 2% agarose gel to distinguish between 175 bp band produced by the digestion of A allele and the 141 bp produced by the digestion of the G allele. Heterozygous state yielded both 141 bp and 175 bp bands (Figure 1). 

*Statistical Methods*

The statistical analysis of the data was performed by using excel (Microsoft office 2013) program and SPSS (Statistical Package For Social Science) program (SPSS, Inc, Chicago, IL) version 20. A Chi-square test was used to compare groups. Quantitative data were presented as median and range, mean and standard deviation. For comparison between two groups, student T-test (for parametric data) and Mann-Whitney test (for non-parametric data) were used. For comparison of more than two groups; One-Way ANOVA (for parametric information) and Kruskal-Wallis (for non-parametric data) were used. For comparison within the same group, paired-sample T-test (for parametric data) and Wilcoxon test (for non-parametric data) were used. The univariate analysis used to evaluate the association of polymorphism with the occurrence of leukemia and hepatotoxicity. Multivariate logistic regression used for hepatotoxicity. For survival analysis, Kaplan-Meier curves were used and compared by the log-rank test.

## Results

The age of the studied patients ranged from 5 to 18 years. Among the 50 patients, 32 (64%) were males, and 18 (36%) were females. The age of the control group ranged from 3 to 12 years (mean 8.66±3.50). Among the 50 healthy children, 26 (52%) were males and 24 (48%) females. Immunophenotyping of ALL patients revealed 32 (64%) patients B-ALL and 18 (36%) patients T-ALL. 

In the current study, there was a significant elevation of ALT in T ALL patients compared to control (P-value 0.008). On the other hand, there is no change regarding AST between studied groups. There was also a significant elevation of post-treatment AST (P-value 0.011) and ALT (P-value 0.044) compared to pretreatment in T ALL group. However, there is no significant change in B ALL (Table 1).

Comparison of cyclin D1 variant between cases and control revealed that there was significantly higher AA genotype and A allele in acute leukemia patients when compared to the control group (P-value 0.001), while other genotyping (GG and GA) showed no significant differences between cases and controls. Also, there was a real risk of developing ALL with AA genotype, and the results were highly statistically significant for AA genotype compared to the GG genotype. The study also revealed significant risk for AA genotype compared to non-homozygosity for AA genotype (AG+GG) (Table 2).

Furthermore, the effect of cyclin D1 genotype on the incidence of hepatotoxicity after one week of methotrexate administration as an indicator of methotrexate toxicity were assessed. we didn’t find any significant differences between hepatotoxic and non-hepatotoxic patients regarding cyclin D1 genotypes despite a highly significant difference in methotrexate level. (P-value 1.00) (Table 3and4).

Logistic regression analysis was conducted for the prediction of hepatotoxicity in T ALL patients. Age, gender, cyclin D1 genotypes were applied as covariates. None of the covariates were associated with the risk of hepatotoxicity in univariate analysis. (P-value 1.00), (P-value 0.250) and (P-value 1.00) respectively (Table 5). 

No significant differences were found regarding cyclin D1 genotypes in patients with complete remission and partial remission or relapse (Table 6). 

**Figure 2:**
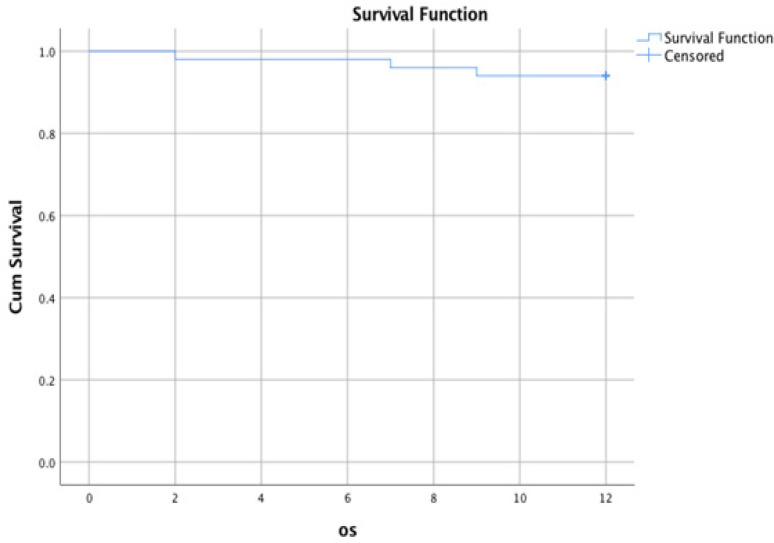
Overall Survival (OS) of Studied ALL Cases

**Figure 3:**
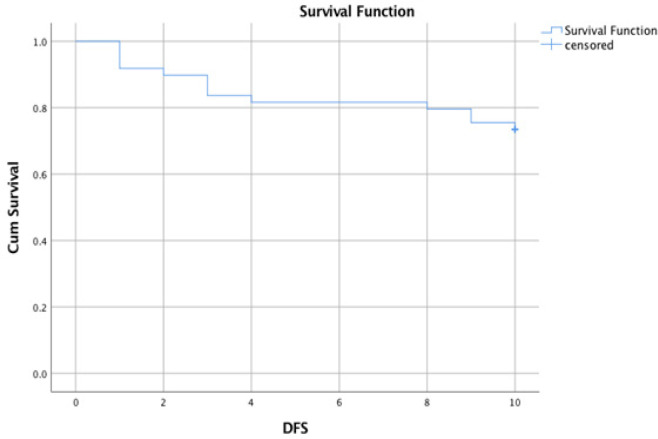
DFS of Studied ALL Cases

**Figure 4:**
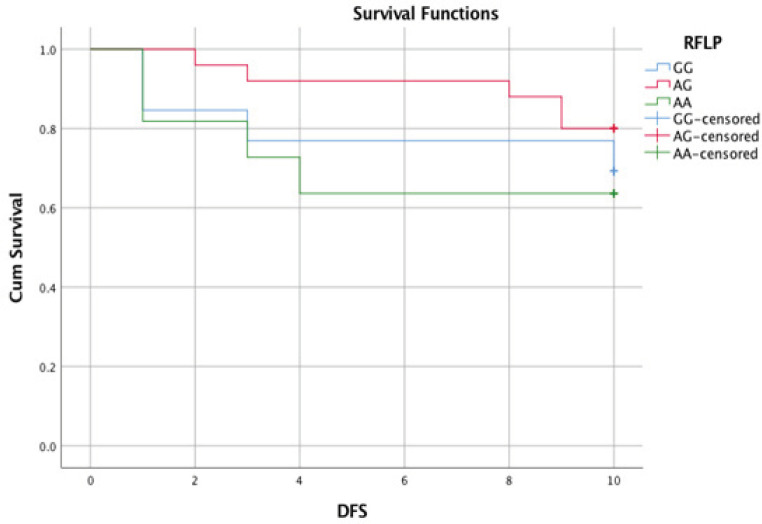
DFS in Relation to cyclin D1 Variant

At the end of the follow-up period (12 months), the OS of studied ALL cases estimates 98% at six months and 96% at 12 months (figure2). As regard disease, free survival was also rated 82% at five months and 74% at ten months (figure 3). As regards GG genotype DFS was estimated 78% at five months and 68% at ten months. Also, AG genotype was determined 92% at five months and 80% at ten months. On the other hand, AA genotype was estimated at 64% at five months and 64% at ten months, with no significant difference between 3 genotypes (P-value 0.474) (Figure 4).

**Table 1:** Comparison of Liver Enzymes in Case Groups before and after Treatment

	Parameter	Pretreatment			Post-treatment			P
		Median	Range		Median	Range		
			Min	Max		Min	Max	
B ALL	AST	20	4	141	29	15	45	0.896
	ALT	31	12	98	29	12	45	0.071
T ALL	AST	30	8	129	93	12	1567	0.011
	ALT	39	1	127	85	8	1258	0.044

Wilcoxon test*; *significant (P-value < 0.05)

**Table 2:** Comparison of Cyclin D1 Variant between Cases and Control

Control (n=50)		Acute leukemia (n=50)	P	OR	95%CI	
GG		13 (26%)	0.002			
AG		25 (50%)		1.846	0.77	4.421
AA	1 (2%)	12 (24%)		22.154	2.584	189.958
AG+AA	26 (52%)	37 (74%)		2.627	1.133	6.092
AG+GG	49 (98%)	38 (76%)		15.47	1.92	124.3
AA	1 (2%)	12 (24%)				
G	73 (73%)	51 (51%)		2.597	1.439	4.688
A	27 (27%)	49 (49%)				

Chi-Square test, odds ratio, CI confidence interval. all data in tables presented as count and percentage; **significant (P value < 0.05); P value of chi square test result from comparing 3 genotypes between two studied groups.

**Table 3:** Comparison of Cyclin D1 Variant between Patients with and without Hepatotoxicit

Without hepatotoxicity (n=7)		Hepatoxicity (n=10)	P	OR	95%CI	
GG	1 (14.3%)	3 (30%)	0.442			
AG	5 (71.4%)	4 (40%)		0.267	0.019	3.653
AA	1 (14%)	3 (30%)		1	0.041	24.547
AG+AA	6 (85.7%)	7 (70%)		0.388	0.031	4.795
AG+GG	6 (85%)	7 (70%)		2.571	0.208	31.71
AA	1 (14.3%)	3 (30%)				
G	7 (50%)	10 (50%)		1	0.255	3.91
A	7 (50%)	10 (50%)				

Odds ratio , CI confidence interval. all data in tables presented as count and percentage; **significant (P value < 0.05); P value of chi square test result from comparing 3 genotypes between two variants.

**Table 4:** Comparison of Methotrexate Level at 48 Hours after Administration between Patients with and without Hepatotoxicity

Parameter	Without hepatotoxicity (n=7)			Hepatoxicity (n=10)			P
	Median	Range		Median	Range		
		Min	Max		Min	Max	
Methotrexate (umol/L)	0.5	0.2	0.8	2.75	1.9	4	0.001

Mann-whitney tests. P between two groups; **significant (P value < 0.05)

**Table 5:** Logistic Regression Analysis for Prediction of Hepatotoxicity within T ALL Patients

Parameter		No hepatotoxicity	Hepatotoxicity	Univariate			
				OR	95% CI		P
Age	<5years	1	2	0.667	0.048	9.189	1
	>5 years	6	8				
Gender	Male	4	9	0.148	0.012	1.9	0.25
	Female	3	1				
Cyclin D1 genotype	GG	1	3				
	AG	5	4	0.267	0.019	3.653	0.308
	AA	1	3	1	0.041	24.547	1

**Table 6:** Comparison of Cyclin D1 Variant between Partial Remission or Relapse and Complete Remission

		Complete remission (n=35)	Partial remission or relapse (n=14)	P	OR	95%CI	
GG	Count, %	9 (25.7%)	4 (28.6)	0.987			
AG	Count, %	18 (51.4%)	7 (50%)		0.875	0.202	3.791
AA	Count, %	8 (22.9%)	3 (21.4)		0.844	0.143	4.974
AG+AA	Count, %	26 (74.3%)	10 (71.4%)		0.865	0.216	3.458
AG+GG	Count, %	27 (77.1%)	11 (78.5%)		0.920	0.205	4.128
AA	Count, %	8 (22.9%)	3 (21.5%)				
G	Count, %	36 (51.4%)	15 (53.6%)		0.917	0.381	2.208
A	Count, %	34 (48.6%)	13 (46.4%)				

P value of chi square test result from comparing 3 genotypes between two; **significant (P value < 0.05)

## Discussion

Acute lymphoblastic leukemia (ALL) is the most common cancer in children, and although the cure rates exceed 90%, it remains a significant cause of morbidity and mortality in children and adults. The past decade has been marked by extraordinary advances in the genetic basis of leukemogenesis and treatment responsiveness in ALL (Iacobucci andMullighan, 2017).

Till now, over 50 years have gone after MTX was first presented in the clinic. It has been involved in most of the treatment protocols of many solid tumors, including hematological malignancies, including ALL. High-dose methotrexate (HDMTX) refers to infused MTX in doses of more than 1 g/m^2^. Using HDMTX shows the marvelous result in treating ALL and prevention of extramedullary leukemia (i.e., central nervous system leukemia and testicular leukemia), as MTX can penetrate the blood-brain barrier and blood-testis barrier (Qui et al.,2017.

The primary target of MTX is the enzyme dihydrofolate reductase (DHFR), which can catalyze the reduction of folate and 7, 8-dihydrofolate to 5, 6, 7, 8-tetrahydrofolate. This will lead to the impairment of nucleic acid synthesis and cellular death. Many proteins involved in the folate metabolic pathway (i.e., MTHFR, RFC, TS) may contribute to MTX cytotoxicity and clinical elimination (Liu et al.,2017). 

Cyclin D1 is involved in the folate metabolic pathway and has been shown to influence cellular response to MTX. Thus CCND1 influences the clearance of MTX and MTX-associated toxicity partly through the regulation of DHFR and THF (Xue et al., 2015).

The present study revealed a highly significant association between A allele either in heterozygous (AG) or homozygous state (AA) and cases. Besides, there was a real risk of developing ALL with AA genotype, and the results were highly statistically significant for AA genotype compared to the GG genotype. These findings were concordant with those of Hou et al., (2005), who studied 183 ALL cases and 190 age and sex-matched healthy controls, and found that CCND1 A allele was more frequent in the ALL group than in the control group. Hou et al., (2005) and Bedewy et al., (2013) who studied the association of *CCND1 G870A *polymorphism with ALL risk in 25 ALL patients and 15 healthy controls and found that frequency of the AA genotype was significantly increased in the ALL cases while GG genotype was significantly increased in the control group. In more addition significant association between *CCND1 G870A *polymorphism and Nasopharangyl carcinoma risk was found in the central-southern Chinese population (Liao et al., 2014).

The study also revealed significant risk for AA genotype when compared to non-homozygosity for AA genotype (AG+GG) in contrast to Hou et al., (2005) and Bedewy et al., (2013). The impact of the CCND1 A allele on childhood ALL was thought to be due to a higher level of CCND1b resulting in a longer half-life protein, which might affect the transition from the G1 to S phase of cell cycle and in turn cell proliferation (Knudsen, 2006)

Because ALL patients were administrated with MTX in different doses (5.0 g/m^2^ for children with high-risk or medium-risk ALL and 3.0 g/m^2^ for children with low-risk ALL), a stratification analysis was conducted.

Relations between clinical response and systematic toxicity after MTX treatment and gene variation in the folate metabolic pathway have been detected many times Most studies have focused on MTHFR C677T and RFC G80A and suggested that these polymorphisms contribute to MTX therapy-related toxicity or outcome of childhood ALL (Umerez et al., 2017, Tiwari et al., 2018).

In this study, we studied the effect of cyclin D1 genotype on the incidence of hepatotoxicity after one week of methotrexate administration as an indicator of methotrexate toxicity. We didn’t find any significant differences between hepatotoxic and non-hepatotoxic patients regarding cyclin D1 genotypes. 

The results of the current study were matched with results of Dulucq et al., (2008) who did not find any association between toxicity parameters investigated and the increasing number of event-predisposing genotypes DHFR haplotype*1, CCND1 870AA, and TS 3R3R. But they thought this was most likely because in these three at-risk genotypes, only the CCND1, but not TS and DHFR, were individually associated with a lower frequency of toxic events (Dulucq et al., 2008). 

However, in contrast with the results of the present study, Costea et al. (2006) observed that individuals with CCND1 870AA genotype had lower liver toxicity rates. They also found significantly lower rates of these toxicities in individuals with a combination of the CCND1 870AA and MTHFR 677 TT/CT genotypes. This observation may be explained by the variant isoform of CCND1 with a longer half-life and the correlated higher level of DHFR (Costea et al., 2006). 

Furthermore, Xue et al., (2015) have found that subjects with 870AG and 870AA genotypes have significantly higher rates of MTX-related hepatotoxicity. They also found a statistically significant relationship between the hepatotoxicity and the combined genotypes AG/AA, suggesting that the 870A allele may be a risk factor of toxic events. Their subgroup analysis showed that the association of *G870A* polymorphism and an increased risk of MTX-related hepatotoxicity was more pronounced in children administrated with MTX in a dose of 5.0 g/m2 (high-risk ALL and medium risk ALL). This discrepancy may be due to different genetic backgrounds and population-specific differences (Xue et al., 2015). 

After following up ALL patients for one year, there was no statically significant difference among CCND1 genotypes regarding overall survival and event-free survival (P-value 0.474). However, there was a clinically significant association between CCND1 AA genotype and poor survival outcome compared to AG and GG genotypes in contrast to Costea et al., (2006) who studied 205 children of French-Canadian origin with ALL and follow them up for 60 months, the analysis of the impact of CCND1 showed that the individuals with AA variant had a remarkably lower probability of 5-year post-treatment EFS compared to those with AG and GG genotypes (37% compared to 88%, P< 0.00005).

Moreover, some other confounding factors may interfere with the present results, such as some unknown gene variations in the folate metabolic pathway. At the same time, the fact cannot be ignored that our chemotherapy regimen is not identical to other research institutions ( Dulucq et al., 2008; Kantar, 2009) and some chemotherapeutic drugs used in our center may also influence the evaluation of MTX-related hepatotoxicity. 

We are studying genetic factors aid in early diagnosis and treatment of ALL. CCND1 AA genotype and A allele are associated with high risk to develop ALL.* Cyclin D1 *polymorphism may not be contributed to MTX induced hepatotoxicity and couldn’t be useful in the clinical prediction of prognosis.

The sample size was relatively small, so it led to a larger span of 95% CI (0.041 – 24.547). Further studies with a large sample size are needed to confirm the relationship between gene polymorphism and MTX induced hepatotoxicity. In the present study, one laboratory index was only explored (serum aminotransferase). In further studies, other toxicities such as oral mucositis and vomiting should be observed, and the Serum level of MTX should be involved to assess MTX clearance association with CCND1 genotype.
